# Does response to vagus nerve stimulation for drug‐resistant epilepsy differ in patients with and without Lennox–Gastaut syndrome?

**DOI:** 10.1002/brb3.3025

**Published:** 2023-06-29

**Authors:** Maxine Dibué, Teresa Greco, Jochem K. H. Spoor, Christian Senft, Marcel A. Kamp

**Affiliations:** ^1^ Medical Affairs International, Neuromodulation LivaNova PLC London UK; ^2^ Statistics and Data Management LivaNova PLC London UK; ^3^ Department of Neurosurgery Erasmus University Rotterdam The Netherlands; ^4^ Department of Neurosurgery, Medical Faculty Friedrich Schiller University Jena Germany

**Keywords:** epileptic encephalopathy, Lennox–Gastaut syndrome, seizure type, vagus nerve stimulation

## Abstract

**Introduction:**

Literature on outcomes of patients with Lennox–Gastaut syndrome (LGS) receiving adjunctive vagus nerve stimulation (VNS) lacks information on seizure types and the time course of therapeutic effects. We have therefore performed what is to our knowledge the largest and most in‐depth analysis of the effectiveness of VNS in LGS patients paying special attention to the impact of VNS Therapy on individual seizure types.

**Methods:**

The VNS Therapy Outcomes Registry includes over 7000 patients. A propensity score matching method was employed to match patients with LGS to non‐LGS patients with drug‐resistant epilepsy (DRE). Overall seizure frequencies were assessed prior to implantation and at 3‐, 6‐, 12‐, 18‐, and 24‐month follow‐ups to derive the main study outcomes: response rates and time to first response.

**Results:**

A total of 564 LGS patients with sufficient data were identified in the registry and matched 2:1 to 1128 non‐LGS patients. Responder rates at 24 months were 57.5% in the LGS group and 61.5% in the non‐LGS group. Median seizure frequency reduction at 24 months was 64.3% versus 66.7% in the LGS versus non‐LGS group, respectively. In both groups, VNS was most effective at reducing focal aware seizures, “other” seizures, generalized‐onset non‐motor seizures, and drop attacks with relative reduction rates for these seizure types at 24 months exceeding 90% in both groups. Time‐to‐first response did not differ between the groups; however, there was a significantly higher proportion of patients who regressed from bilateral tonic–clonic (BTC) seizure response in the LGS group versus the non‐LGS group at 24 months: 22.4% versus 6.7%; *p* = .015.

**Conclusions:**

Although limited by its retrospective design, the study shows that the effectiveness of VNS is comparable in DRE patients with and without LGS; however, LGS patients may be more prone to fluctuating control of BTCs.

## Graphical abstract

At 24 months of VNS, responder rates were 57.5% vs 61.5% in the LGS vs non‐LGS group, respectively. VNS was most effective at reducing the same seizure types in both groups. LGS patients displayed more fluctuating control of bilateral tonic‐clonic seizures

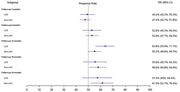

## 1 INTRODUCTION

Lennox–Gastaut syndrome (LGS) is a severe epileptic encephalopathy of childhood‐onset and poses a therapeutic challenge to physicians (Cross et al., 2017). By combining and balancing multiple therapeutic options to treat seizures, the goal is to prevent negative effects on comorbidities with the best possible behavioral and neurodevelopmental outcomes (Strzelczyk & Schubert‐Bast, 2021). These treatments can be pharmacological or non‐pharmacological and must address multiple seizure types in a highly drug‐resistant population.

Vagus nerve stimulation (VNS) was approved as an adjunctive therapy for adults and children of all ages with drug‐resistant seizures in 1994 in Europe, and for adults and children over 12 years of age with drug‐resistant focal‐onset seizures in 1997 in the United States (US). The age limitation in the US was later reduced to children over 4 years of age.

VNS Therapy involves intermittent electrical stimulation of the left cervical vagus nerve. This stimulation induces action potentials traveling predominantly afferently to the brain where they modulate the metabolism and excitability of structures that compose what is being increasingly referred to as the vagal afferent network (Hachem et al., 2018). VNS Therapy is generally considered when patients with drug‐resistant epilepsy (DRE) are not candidates for resective surgery, either for etiological reasons, patient preference, or caregiver preference. Typically, with adjunctive VNS Therapy, approximately 60% of DRE patients experience 50% or more seizure frequency reduction, whereas 40%–50% of patients with DRE experience a reduction in seizure severity or duration. Improvements in mood and certain domains of cognition have also been shown to be associated with VNS Therapy (Orosz et al., 2014; Spindler et al., 2019).

Numerous investigations of VNS Therapy in patients with LGS have been published in the past 25 years; many of them report similar response rates for LGS patients and heterogeneous DRE populations (Dibué et al., 2021). However, the majority of the studies do not report the effects of VNS Therapy on individual seizure types. One study investigating the effects of VNS and callosotomy in LGS patients found both treatments to be effective in controlling atypical absences and bilateral tonic–clonic (BTC) seizures; VNS to be less effective than callosotomy in reducing drop seizures, and more effective than callosotomy in reducing myoclonic seizures; and both treatments to be ineffective in controlling tonic seizures. However, this was the experience of 44 patients at a single center, and these results have yet to be reproduced in larger cohorts (Cukiert et al., 2013). Our recently published analysis in which demographics and clinical characteristics of DRE patients with and without LGS who later underwent VNS implantation were compared found that prior to VNS, LGS patients were taking more anti‐seizure medications (ASMs) with poorer seizure control and had more than twice the seizure burden than non‐LGS patients with mainly BTCs contributing to this difference (Spoor et al., 2021). Approximately 11% of LGS patients had undergone prior epilepsy surgery compared to 19% of non‐LGS patients. Considering these differences in disease burden, the question arises whether there are also differences in VNS response among the groups. We therefore performed what is to our knowledge the largest and most in‐depth analysis of the effectiveness of VNS in LGS patients paying special attention to the impact of VNS Therapy on individual seizure types and on the time course of response.

## 2 MATERIALS AND METHODS

### 2.1 Study design

This is a post‐market registry‐based prospective cohort study of patients diagnosed with DRE and treated with VNS Therapy adjunctive to ASMs, which uses propensity score matching (PSM) to compare outcomes of two patient groups within the registry. The design and analysis of this study were conducted in accordance with the Strengthening the Reporting of Observational Studies in Epidemiology and the Reporting of Studies Conducted using Observational Routinely‐Collected Health Data statements (Benchimol et al., 2015; Von Elm et al., 2007). This retrospective analysis was approved by the ethical committee of the Friedrich Schiller University Hospital (2022‐2575).

### 2.2 Setting and participants

The VNS Therapy Patient Outcome Registry includes patients receiving VNS Therapy as an adjunctive treatment for DRE. The registry was established in 1999 by the device manufacturer Cyberonics, Inc. (now LivaNova PLC) after the approval of adjunctive VNS Therapy in the US for DRE. The goal of the registry was to systematically monitor treatment outcomes in implanted patients.

### 2.3 Data sources and variables

The registry data were prospectively and voluntarily provided by 1285 prescribing physicians from 978 centers of which 911 were in the US and Canada and 67 in other countries across the world. The physicians or their designated clinical staff completed standard case report forms based on a patient's medical history or current visit and voluntarily submitted the forms to the registry for data entry. Previously, investigators have authenticated the integrity of the systems for collecting and processing registry data using an independent auditing agency (Amar et al., 2004).

All study data were de‐identified prior to analysis. Individual, de‐identified data were only used to construct aggregate statistics, including age‐standardization. Only aggregate data were retained and presented.

Access to data was restricted to a minimum number of study investigators and accessed via encrypted security codes without further distribution prior to de‐identification.

The database was queried to extract seizure outcomes reported at baseline and up to 24 months after VNS implantation, as well as safety data of patients with DRE with or without LGS.

### 2.4 Bias

The PSM homogeneous population was identified to control for selection bias. A propensity score method was used to match patients in the disease populations, that is, LGS patients with DRE and non‐LGS patients with DRE (Rosenbaum & Rubin, 1983). An ordinal logistic regression model was run to regress the disease population variable on age at implant, age at diagnosis, and sex. Patients were paired based on an optimal matching with a 1:2 ratio assuming that the number of LGS patients in the registry is at least half of the number of non‐LGS patients.

### 2.5 Sample size

This is an enumerative study, and the sample size is not based on a statistical power calculation. The eligibility of all patients in the VNS Therapy Patient Outcome Registry was evaluated for inclusion in the analysis.

### 2.6 Statistical methods

Demographics and de‐identified patient characteristics were analyzed by means of summary statistics, as appropriate, for continuous or categorical variables.

Seizure counts were investigated overall and by seizure type. The registry's case reports form collected data on the frequency of the following seizure types according to the International League Against Epilepsy (ILAE) seizure classification from 1981: simple partial, complex partial, generalized tonic–clonic, secondary generalized, absence, drop attack, and aura. Here, we refer to the seizure types according to the ILAE seizure classification from 2017 (Fisher et al., 2017): focal aware (FA) motor, focal impaired awareness (FIA) motor, BTC, focal‐to‐BTC (FBTC), generalized onset non‐motor (GONM) with the exception of drop attack, which may comprise multiple seizure types that lead the patient to fall such as generalized onset atonic or BTC. Analysis of the seizure type “aura” was discontinued owing to a low sample size delivering unreliable data.

Analyses by seizure type were performed in patients having a seizure count of >0 for a specific seizure type at baseline.

Subjects with a new seizure type onset were not counted to evaluate changes from baseline. At each post‐baseline visit, a continuity correction was applied to 0 seizure counts becoming 0.5. However, the number of patients for whom a seizure type was reported at a follow‐up visit that was not reported during the baseline period was assessed per seizure type.

The primary endpoint in this study was the seizure response rate at each available follow‐up assessment in LGS patients with DRE versus matched non‐LGS patients with DRE included in the PSM population (hereafter referred to as the LGS group and the non‐LGS group, respectively). Responder status was defined as a reduction in seizure frequency from the baseline of 50% or more. A regressed status was defined as a subject who was in responder status at the previous follow‐up visit but had less than 50% seizure frequency reduction compared to baseline at a later follow‐up visit.

At each time point, the proportions of responders and of regressed subjects, together with the corresponding 95% confidence intervals (95% CI), were derived from the Clopper–Pearson method.

Independently, for each follow‐up, a two‐proportion *z*‐test was applied to compare the responder rates between the LGS group and the non‐LGS group. As this is a non‐confirmatory study, no approaches were considered to correct for multiplicity.

Missing data on the primary endpoint were imputed according to missing at random assumption and taking into consideration specific baseline characteristics, that is, age at implant, age at diagnosis, sex, average seizure count at baseline, and seizure type at baseline. Two‐hundred imputation datasets were generated and combined for the inference using the Little and Rubin framework (Little & Rubin, 1987). Distribution of average count of seizures per month and change from baseline in seizure counts were summarized descriptively by LGS and non‐LGS populations. Due to the non‐normal distribution of the seizure differences, the median of changes from baseline (i.e., calculated as the median of all differences between the seizure count at the follow‐up visit and at baseline) was provided together with the 95% CI derived by the bootstrap method (Puth et al., 2015). The Wilcoxon test was used to compare the medians of the two groups.

Generalized Estimating Equations (GEE) models assuming (i) a binomial distribution with a logit link function for the responder rate outcome and (ii) a negative binomial distribution with a log‐linear link function for the post‐implant average seizures counts were run overall and by seizure type to evaluate the effect of the disease populations (LGS vs. non‐LGS) (Liang & Zeger, 1986). The models were adjusted for the effect of the follow‐up. The exchangeable correlation was assumed within‐patients.

Kaplan–Meier plots and estimates for time‐to‐first response were provided for each disease population. The starting date of the survival functions was from the study day 0 (implant date). Time was in months until termination, and it was censored at the date of discontinuation or analysis cutoff date.

As our baseline analysis indicated a higher proportion of patients with developmental delay and mental retardation in the LGS group, safety endpoints were chosen that are less biased by the impairment of patients to report them (e.g., hoarseness in a nonverbal patient). Therefore, safety was evaluated based on reported hospitalizations and all‐cause mortality obtained from the total patient years of exposure during the registry period.

## 3 RESULTS

### 3.1 Demographics and clinical characteristics

The VNS Therapy Patient Outcome Registry included a total of 7383 patients. Of these patients, 808 (10.9%) had an LGS diagnosis and 6575 (89.1%) had an alternative diagnosis. The full analysis set included 7311 patients who had at least 1 value on overall seizure count and seizure type count: A total of 645 (11%) were in the LGS group, and 5288 (89%) were in the non‐LGS group. The propensity score method selected a cohort of 1692 patients homogeneous for age at implant, age at diagnosis, and sex between the LGS group (564 [33.3%]) and the non‐LGS group (1128 [66.7%]) (Table S1). The demographics and clinical characteristics of the two groups in the PSM population at baseline (including patients without an optimal matching or sufficient seizure data that are excluded in this analysis) have been previously described in detail by Spoor et al. (2021).

At 3, 6, 12, 18, and 24 months of follow‐up, the numbers of patients included in the analysis in the LGS group were 463, 299, 276, 178, and 133, and the numbers of patients in the non‐LGS group were 892, 613, 549, 333, and 195.

### 3.2 Response rates

**FIGURE 1:**
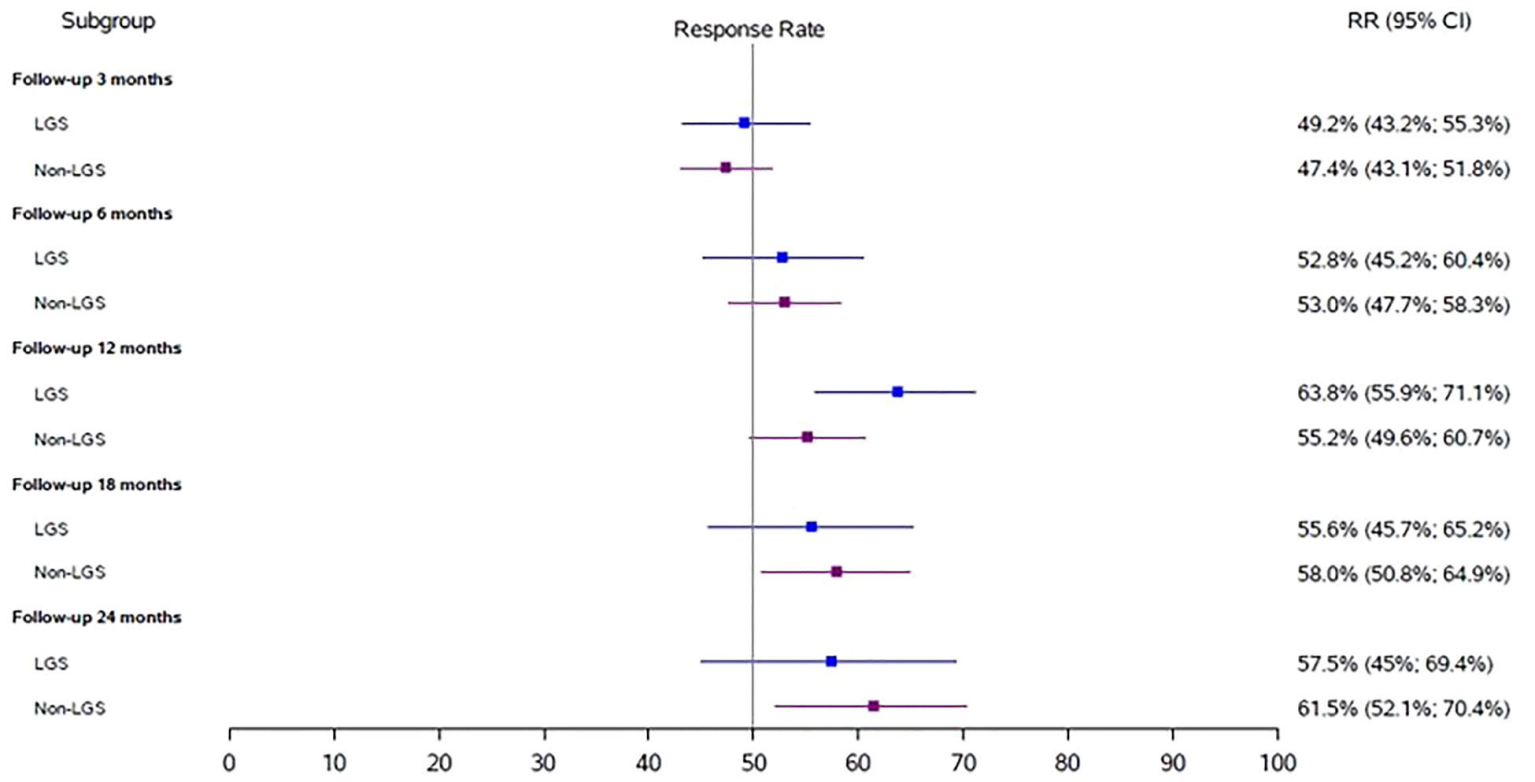
**Response rate based on all seizure types**: Forest plot for response rates based on all seizures for both groups. CI, confidence intervals; LGS, Lennox–Gastaut syndrome.

At 3, 6, 12, 18, and 24 months, the respective responder rates based on total seizures (95% CI) were largely the same for the LGS group: 49.2% (43%, 55%), 52.8% (45%, 60%), 63.8% (55.9%, 71.1%), 55.6% (46%, 65%), and 57.5% (45%, 70%) and the non‐LGS groups: 47.4% (43%, 52%), 53% (48%, 58%), 55.2% (49.6%, 60.7%), 58% (51%, 65%), and 61.5% (52%, 70%) (Figure 1). Responder rates significantly differed at 12 months: 63.8% (56%, 71%) for LGS and 55.2% (50%, 61%) for non‐LGS (unadjusted *p* = .019). When missing data were imputed, responder rates did not significantly differ between the groups at any time point and were: 48.1% (44%, 52%) and 46% (36%, 57%) for LGS and non‐LGS populations at 24 months, respectively.

In the LGS group, the median reduction of total seizures was 48.6%, 52.1%, 66.7%, 56.4%, and 64.3% at 3, 6, 12, 18, and 24 months, respectively, compared with 45.5%, 50%, 60%, 61.4%, and 66.7% in the non‐LGS group. The Wilcoxon test comparing the medians of the two groups did not find significant differences in total seizure frequency reduction at any of the follow‐up time points.

The prevalence of individual seizure types showed a different distribution between the two groups. BTCs were the most prevalent seizure type in the LGS group affecting more than half of patients at baseline, whereas BTCs only occurred in a third of non‐LGS patients, in whom FIAs were the most common seizure type at baseline. When comparing the baseline visit to the 24‐month follow‐up visit, no significant changes in seizure type prevalence were observed in the non‐LGS group, whereas FAs were completely eradicated, and the prevalence of “other” seizures were reduced by 15% in the LGS group (Figure S1).

**FIGURE 2:**
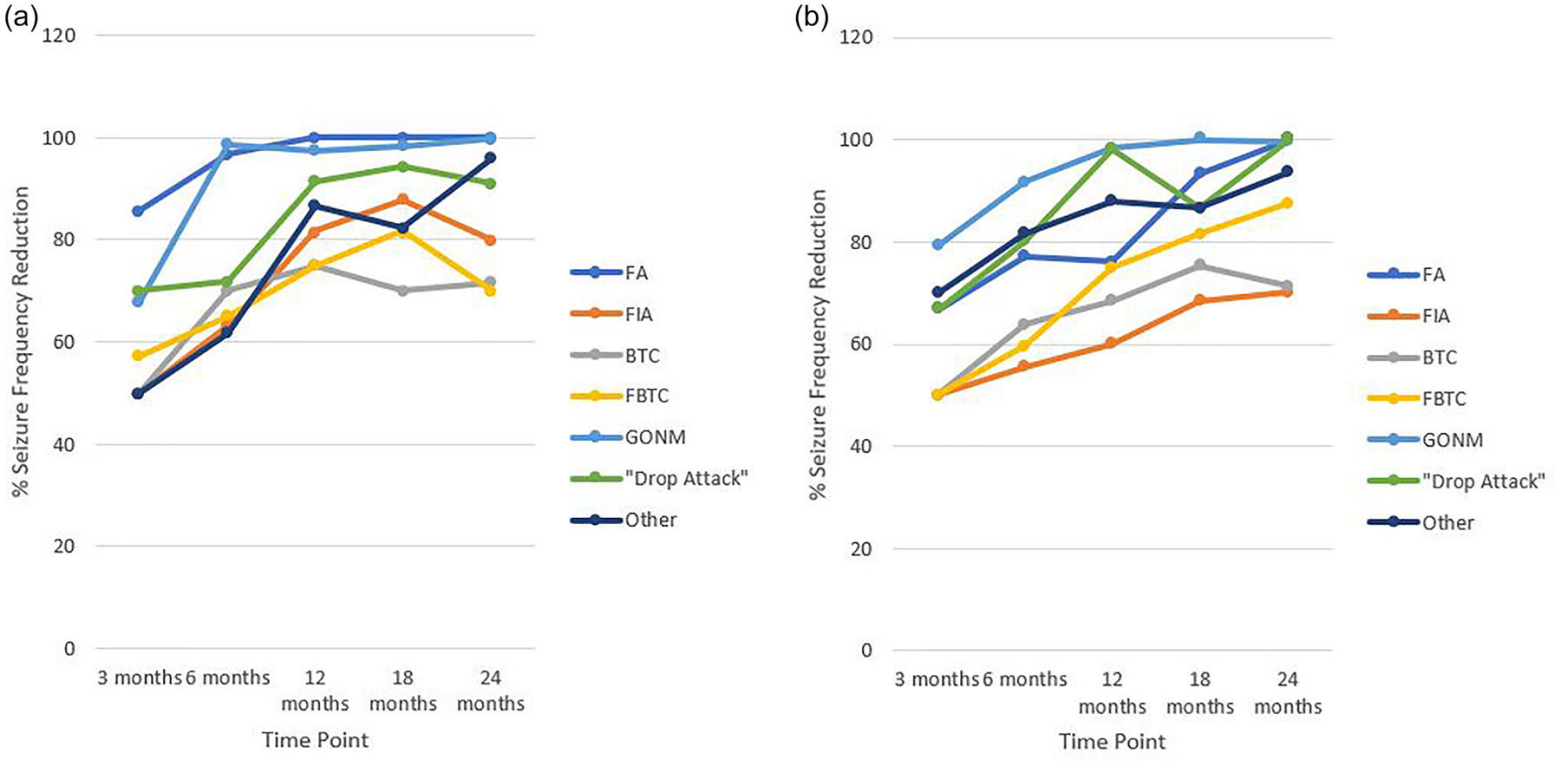
**Reduction of seizures by seizure type**: (A) relative change in seizure frequency by seizure type within Lennox–Gastaut syndrome (LGS) drug‐resistant epilepsy (DRE) patients experiencing that seizure type at baseline; (B) relative change in seizure frequency by seizure type within non‐LGS DRE patients experiencing that seizure type at baseline. BTC, bilateral tonic‐clonic; FA, focal aware; FBTC, focal‐to‐BTC; FIA, focal impaired awareness; GONM, generalized onset non‐motor.

The Wilcoxon test comparing the medians of both groups did not identify any statistically significant differences in the relative reduction of any individual seizure type at 24 months. In both groups, VNS was most effective at reducing FAs, “other” seizures, GONMs, and “drop attacks” with relative reduction rates for these seizure types at 24 months exceeding 90% in both groups. The median relative reduction for FIAs, BTC, and FBTC at 24 months was 80%, 71.7%, and 70% in the LGS group and 70.2%, 71.4%, and 87.5% in the non‐LGS group, respectively (Figure 2).

As relative changes in seizure frequency of individual seizure types can only be assessed in patients who experienced the seizure type at baseline, it is important to also analyze how many patients reported an emergence of an individual seizure type that they did not report at baseline.

**FIGURE 3:**
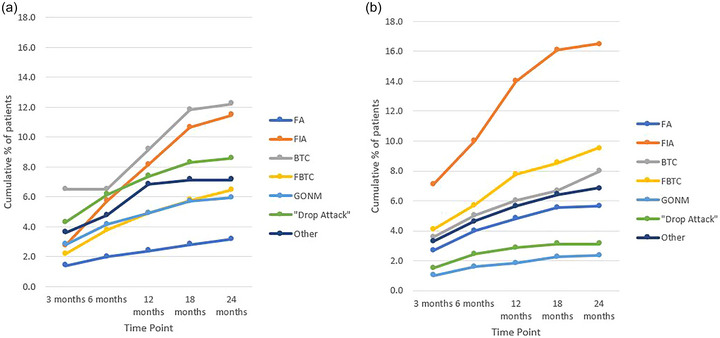
**Rates of emergence of seizure types not reported at baseline**: (A) cumulative rate of emergence of a specific seizure type not reported during the baseline period in the Lennox–Gastaut syndrome (LGS) drug‐resistant epilepsy (DRE) group; (B) cumulative rate of emergence of a specific seizure type not reported during the baseline period in the non‐LGS DRE group. BTC, bilateral tonic‐clonic; FA, focal aware; FBTC, focal‐to‐BTC; FIA, focal impaired awareness; GONM, generalized onset non‐motor.

Cumulative rates of patients who experienced an emergence of an individual seizure type that was not reported at baseline are provided in Figure 3. BTCs, FIAs, and drop attacks were the most frequently emerging seizure types in the LGS group with cumulative rates of 12.2%, 11.4%, and 8.6% at 24 months, respectively. In the non‐LGS group FIAs, FBTCs, and BTCs were the most frequently emerging seizure types with cumulative rates of 16.5%, 9.5%, and 8% at 24 months, respectively.

The highest responder rates by seizure type were for drop attacks (78% and 90% in LGS DRE and non‐LGS groups, respectively) and for FBTCs (74.1% and 73.2% in LGS and non‐LGS groups, respectively) (Figure 4). No differences in time‐to‐first response were found between the LGS group (6, 3–20 months) and the non‐LGS (6, 3–18 months).

FIGURE 4
**Responder rates by seizure type**: (A) forest plot of responder rates for focal impaired awareness (FIA) seizures for both groups; (B) forest plot of responder rates for bilateral tonic–clonic (BTC) seizures for both groups; (C) forest plot of responder rates for focal‐to‐BTC (FBTC) seizures for both groups; (D) forest plot of responder rates for drop attacks for both groups. CI, confidence intervals; LGS, Lennox–Gastaut syndrome;

**Figure:**
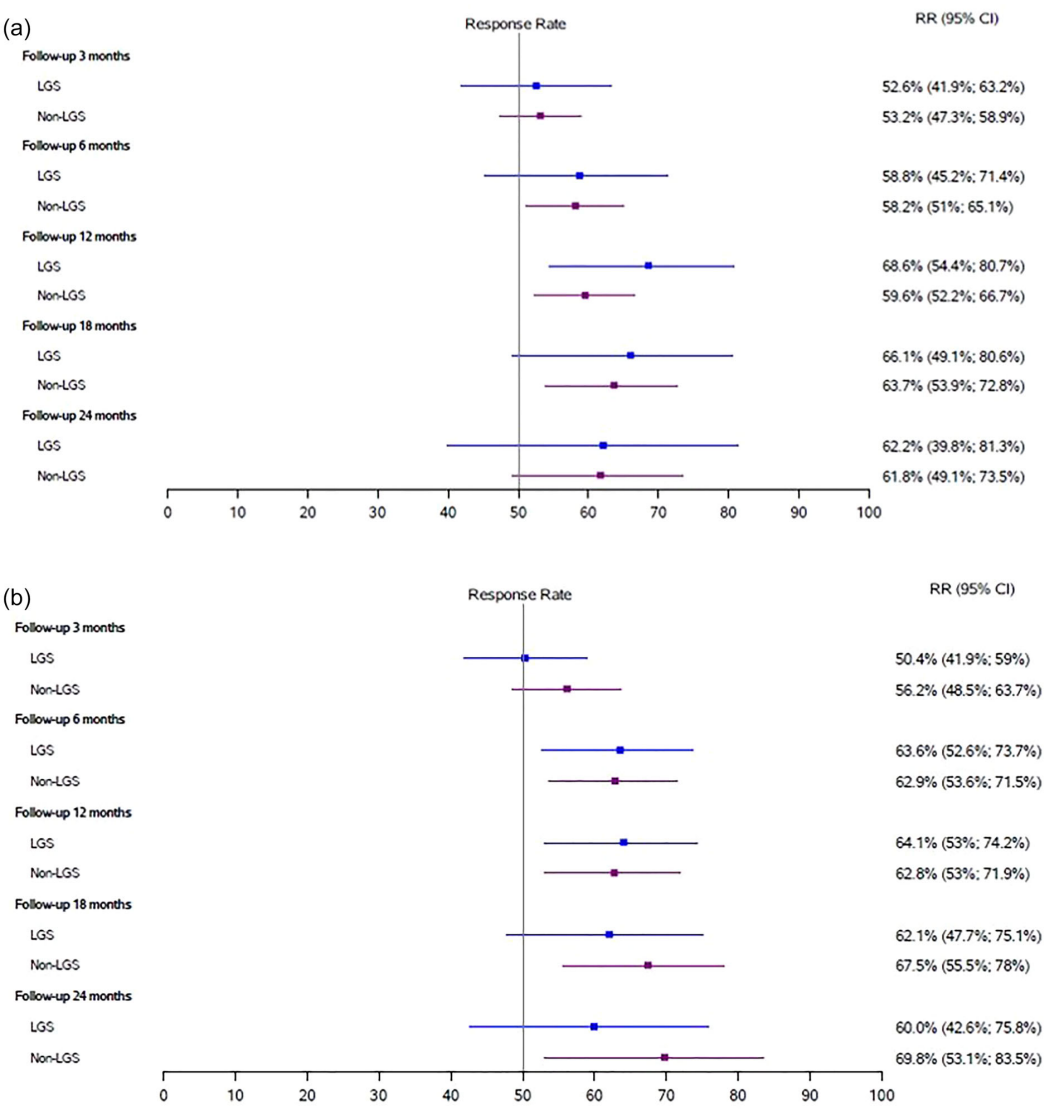

**Figure:**
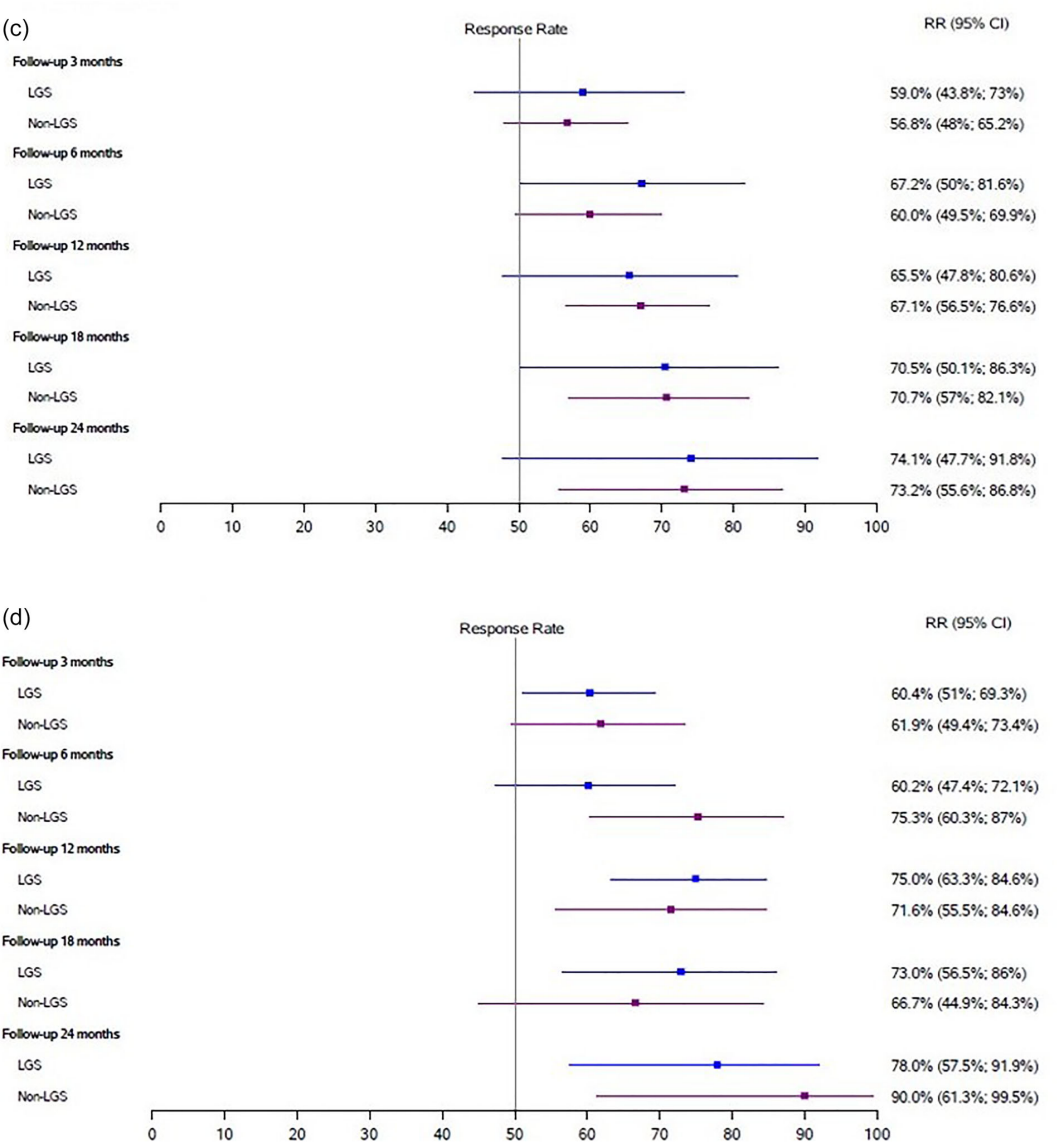

### 3.3 Durability of response

To assess the durability of response to VNS, the proportion of patients who regressed from a state of response to a state of nonresponse (<50% seizure frequency reduction) was analyzed. Although statistical significance was not achieved, there was a trend toward more regression when assessing total seizures in the LGS group versus the non‐LGS group at later follow‐up visits: 6.8% (3.5%–12%) versus 9.7% (6.7%–14%) *p* = .12 at 12 months; 11% (5.8%–19%) versus 8.7% (5.1%–14%) *p* = .4 at 18 months; 15% (7.1%–25%) versus 9.5% (4.8%–16%) *p* = .2 at 24 months, respectively. This trend was driven by the significantly higher number of subjects who regressed from BTC response in the LGS group versus the non‐LGS group at 24 months: 22.4% (10.2%; 39.3%) versus 6.7% (1.1%; 19.5%); *p* = .015.

### 3.4 Safety findings

Within the 24 months of follow‐up, there were 4 deaths in the LGS group and 5 deaths in the non‐LGS group corresponding to all‐cause mortality rates of 8.4 per 1000 patient years in the LGS group and 5.47 per 1000 patient years in the non‐LGS group. Hospitalizations could not be analyzed due to low data quality: Data coverage was less than 5% and decreased with every follow‐up visit leading to an absence of an evaluable amount of hospitalization data.

## 4 DISCUSSION

To our knowledge, this analysis represents the largest and most in‐depth analysis of the effectiveness of VNS in LGS patients. The results demonstrate that the effectiveness of VNS is comparable in DRE patients with or without LGS. In both populations, total seizure frequency reduction and responder rates were 55%–60% at 24 months. This is consistent with our recent meta‐analysis of adjunctive VNS Therapy in patients with LGS that found a responder rate of 54% across studies (Dibué et al., 2021). Furthermore, VNS was most effective in reducing the frequency of the same seizure types (“other” seizures, GONMs, and drop attacks) and did so to a similar degree in both populations.

Differences in the effects of VNS only emerge as a possible trend when looking beyond the usual endpoints of total seizure frequency reduction and responder rate by considering the time course of response. Although the time‐to‐first response for all seizures or any individual seizure type did not differ between the two populations, patients with LGS exhibited a trend to regress more often from response mainly due to BTCs. This analysis cannot answer whether the higher likelihood of fluctuating BTC response in the LGS group reflects an effect of VNS or the known evolution of seizure types over time in LGS. Although the percentage of patients in the LGS group who regressed from response following VNS was less than 20%, to our knowledge, there are no data on 2‐year regression rates from other adjunctive therapies in patients with LGS to compare this finding to.

The present analysis offers insight into the patterns of response to VNS by individual seizure type, which can be considered important for clinicians, patients, and caregivers who are often particularly concerned about a specific seizure type, which is responsible for the majority of patient and caregiver distress. Typically, FBTCs/BTCs and drop attacks are perceived as the most debilitating seizure types for patients with LGS, the former due to their association with sudden unexpected death in epilepsy and the latter due to high risk for injury. This study found VNS to be most effective in reducing the frequency of drop attacks in both populations. In the LGS group, drop attacks were reduced by more than 90% at 24 months and 78% of patients experienced 50% or greater reduction in drop attacks, whereas 74.1% and 60% experienced a 50% or greater reduction in FBTCs and BTCs, respectively. These data may inform a meaningful conversation with patients and their families with regard to expectations on the impact of VNS on these most debilitating seizure types.

Finally, all‐cause mortality in the LGS group was found to be 8.4 per 1000 patient years, which is similar to the recently reported mortality rate of 6.1 per 1000 patient years in patients with confirmed LGS in the United Kingdom. The mortality found in the non‐LGS group of 5.47 per 1000 patient years is also lower than the all‐cause mortality rate of 13.3 per 1000 patient years reported by Ryvlin et al. (2018). Their analysis of 40,443 patients with VNS Therapy (representing 70% of all patients with VNS in the US implanted from 1988 to 2012) comprised 277,661 patient years of follow‐up with a median duration of follow‐up of 7.6 years. Mortality rates for both groups in this analysis were lower than the mortality rate of 15.9 per 1000 patient years reported for patients with childhood‐onset epilepsy who were not in remission (i.e., drug‐resistant) (Sillanpää & Shinnar, 2010).

### 4.1 Limitations

The VNS Therapy Patient Outcomes Registry is a single‐arm, open‐label, observational registry, and although data were collected prospectively, the present analysis was retrospective in nature. Further limitations arise from the design of the registry: Data were collected voluntarily by clinical staff; however, data entry was not monitored in the stringent way it would be in a clinical trial. This may have especially affected the consistency of the classification of seizure types as well as the diagnosis of LGS across the many different sites. Moreover, variability in the combination of ASMs and in stimulation parameters are factors that are not controlled for and that may introduce bias.

Even though the presence of bias was addressed by analyzing a more homogeneous population, the impact of the propensity score match compared to other techniques was not further assessed. Given the large available sample size, the bootstrap algorithm was used to derive the 95% CI of changes from baseline in seizure counts without including a bias correction. Different correlation structures within the GEE models were not assessed. The missing at random assumption along with the baseline characteristics involved in the imputation method was prespecified prior to reviewing any analysis outputs; however, no additional sensitivity analyses to assess the model robustness to the imputed data were performed.

Another important limitation is that data collection of the VNS Therapy Patient Outcomes Registry ceased in 2003 and therefore does not take newer ASMs approved for LGS, such as clobazam, rufinamide, and cannabidiol, and new stimulation paradigms in VNS, such as responsive VNS into account. Therefore, it will be of interest to repeat this analysis in newer prospective VNS registries and compare it to the current analysis to shed light on the real‐world impact of newer ASMs and new stimulation paradigms. An opportunity for this could be the CORE‐VNS registry, which began recruitment in 2018 at 63 sites worldwide (ClinicalTrials.gov Identifier: NCT0352).

## 5 CONCLUSION

VNS is a safe and effective adjunctive treatment for patients with DRE with or without LGS. The present analysis suggests that LGS patients may be more prone to fluctuating control of BTCs; however, it is unclear whether this is a result of VNS efficacy or reflects the known evolution of seizure types over time in LGS. Major differences in response to VNS between the LGS and non‐LGS groups were not identified. VNS was associated with a significant reduction of the frequency of BTCs and drop attacks by more than 70% and 90%, respectively, in both LGS and non‐LGS groups.

## CONFLICT OF INTEREST STATEMENT

Maxine Dibué and Teresa Greco are employees of LivaNova PLC, and Maxine Dibué holds stock options of LivaNova PLC. Jochem K. H. Spoor received a research grant from LivaNova PLC. The other authors have no conflict of interest to disclose.

### PEER REVIEW

The peer review history for this article is available at https://publons.com/publon/10.1002/brb3.3025.

## Supporting information

Figure S1a information

Figure S1b information

Table S1: Demographics of the PSM population

## Data Availability

The data that support the findings of this study are available upon request from the corresponding author. The data are not publicly available due to privacy or ethical restrictions.
