# Supplementary figures and images for: Does response to vagus nerve stimulation for drug‐resistant epilepsy differ in patients with and without Lennox–Gastaut syndrome?
Source: Brain Behav. 2023 Jun 29;13(8):e3025. doi: 10.1002/brb3.3025 (PMC10454273; doi:10.1002/brb3.3025)

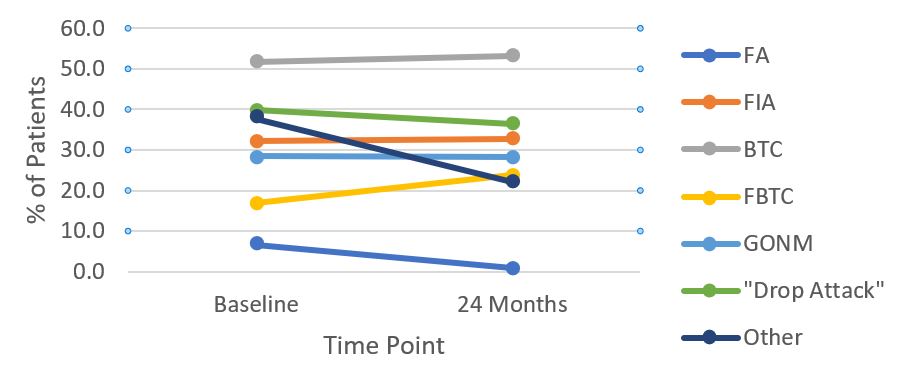

Supplement: Supplementary file 1 — Figure S1a information [file BRB3-13-e3025-s003.JPG]

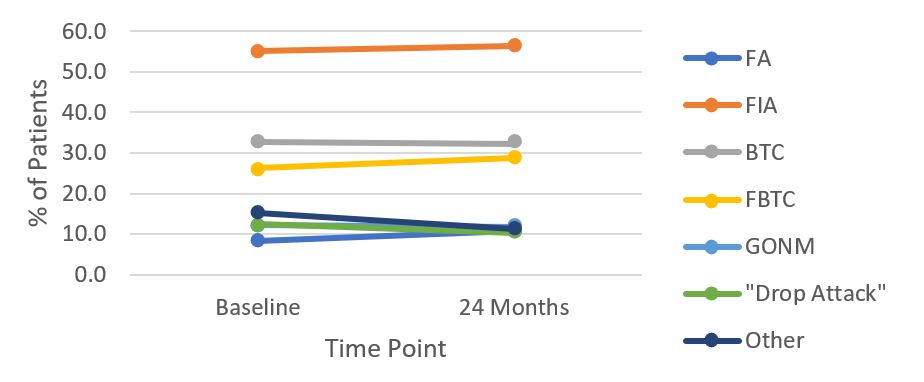

Supplement: Supplementary file 2 — Figure S1b information [file BRB3-13-e3025-s001.JPG]
