# Supplementary material for: Does response to vagus nerve stimulation for drug‐resistant epilepsy differ in patients with and without Lennox–Gastaut syndrome?
Source: Brain Behav. 2023 Jun 29;13(8):e3025. doi: 10.1002/brb3.3025 (PMC10454273; doi:10.1002/brb3.3025)
Supplement: Supplementary file 3 — Table S1: Demographics of the PSM population [file BRB3-13-e3025-s002.docx]

| **Demographics** | | | |
| --- | --- | --- | --- |
|  | **LGS (N=564)** | **non-LGS (N=1128)** | **Total (N=1692)** |
| **Age at implant (year)** |  |  |  |
| Missing | 0 | 0 | 0 |
| N | 564 | 1128 | 1692 |
| Mean ( SD) | 17.8 ( 11.8) | 18.2 ( 12.7) | 18.1 ( 12.4) |
| Median | 14.5 | 15.0 | 15.0 |
| Q1; Q3 | 9; 24 | 9; 25 | 9; 25 |
| Min; Max | 1; 80 | 1; 66 | 1; 80 |
| **Class of age at implant** |  |  |  |
| <1 year | 3 (0.5%) | 10 (0.9%) | 13 (0.8%) |
| 1 - 2 years | 0 | 0 | 0 |
| 3 - 12 years | 12 (2.1%) | 46 (4.1%) | 58 (3.4%) |
| 13 - 17 years | 239 (42.4%) | 468 (41.5%) | 707 (41.8%) |
| => 18 years | 211 (37.4%) | 473 (41.9%) | 684 (40.4%) |
| **Age at diagnosis** |  |  |  |
| Missing | 0 | 0 | 0 |
| N | 564 | 1128 | 1692 |
| Mean ( SD) | 2.2 ( 3.1) | 2.2 ( 3.4) | 2.2 ( 3.3) |
| Median | 1.0 | 1.0 | 1.0 |
| Q1; Q3 | 1; 3 | 0; 3 | 0; 3 |
| Min; Max | 0; 31 | 0; 36 | 0; 36 |
| **Gender** |  |  |  |
| Male | 329 (58.3%) | 631 (55.9%) | 960 (56.7%) |
| Female | 235 (41.7%) | 497 (44.1%) | 732 (43.3%) |

| **Demographics** | | | |
| --- | --- | --- | --- |
|  | **LGS (N=564)** | **non-LGS (N=1128)** | **Total (N=1692)** |
| **Ethnicity** |  |  |  |
| Caucasian | 445 (78.9%) | 940 (83.3%) | 1385 (81.9%) |
| Afro-American | 25 (4.4%) | 64 (5.7%) | 89 (5.3%) |
| Hispanic | 46 (8.2%) | 64 (5.7%) | 110 (6.5%) |
| Asian | 10 (1.8%) | 8 (0.7%) | 18 (1.1%) |
| Other | 14 (2.5%) | 24 (2.1%) | 38 (2.2%) |
| Unknown | 18 (3.2%) | 25 (2.2%) | 43 (2.5%) |
| Not Checked | 4 (0.7%) | 3 (0.3%) | 7 (0.4%) |
| **Weight (kg)** |  |  |  |
| Missing | 57 | 115 | 172 |
| N | 507 | 1013 | 1520 |
| Mean ( SD) | 99.56 ( 50.82) | 110.2 ( 60.01) | 106.6 ( 57.31) |
| Median | 90.00 | 104.0 | 99.00 |
| Q1; Q3 | 57.0; 134.0 | 58.0; 151.0 | 58.0; 146.0 |
| Min; Max | 20.0; 324.0 | 19.0; 364.0 | 19.0; 364.0 |
| **Number of ASMs used at implant** |  |  |  |
| 0 | 3 (0.5%) | 8 (0.7%) | 11 (0.7%) |
| 1 | 50 (8.9%) | 158 (14.0%) | 208 (12.3%) |
| 2 | 198 (35.1%) | 461 (40.9%) | 659 (38.9%) |
| 3 | 221 (39.2%) | 383 (34.0%) | 604 (35.7%) |
| 4 | 65 (11.5%) | 100 (8.9%) | 165 (9.8%) |
| 5 | 27 (4.8%) | 18 (1.6%) | 45 (2.7%) |

| **Demographics** | | | |
| --- | --- | --- | --- |
|  | **LGS (N=564)** | **non-LGS (N=1128)** | **Total (N=1692)** |
| **Number of ASMs used at implant** |  |  |  |
| Missing | 0 | 0 | 0 |
| N | 564 | 1128 | 1692 |
| Mean ( SD) | 3 ( 1.0) | 2 ( 0.9) | 2 ( 0.9) |
| Median | 3 | 2 | 2 |
| Q1; Q3 | 2; 3 | 2; 3 | 2; 3 |
| Min; Max | 0; 5 | 0; 5 | 0; 5 |
